# Tolosa-Hunt Syndrome: A Diagnostic Challenge and the Complexities of Long-Term Corticosteroid Management

**DOI:** 10.7759/cureus.116651

**Published:** 2026-09-21

**Authors:** Guilherme Henriques, Margarida Pistola, Cláudia Resende, Tetyana Yaroshyk, Inês Mezias

**Affiliations:** 1 Family Medicine, Unidade Local de Saúde da Lezíria, Santarém, PRT

**Keywords:** case report, cavernous sinus, corticosteroid-induced hyperglycemia, high-dose corticosteroids, painful ophthalmoplegia, primary care, tolosa-hunt syndrome

## Abstract

Tolosa-Hunt syndrome (THS) is an uncommon cause of painful ophthalmoplegia resulting from idiopathic granulomatous inflammation involving the cavernous sinus, superior orbital fissure, or orbit. Its diagnosis remains challenging because early manifestations may be nonspecific and its clinical and radiological features overlap with several vascular, infectious, inflammatory, and neoplastic conditions. We report the case of a 59-year-old woman with type 2 diabetes mellitus, arterial hypertension, dyslipidemia, and post-thyroidectomy hypothyroidism who initially presented to her family physician with retro-orbital headache and blurred vision, with no focal neurological abnormalities on examination. Her symptoms subsequently progressed to diplopia, complete right-sided ptosis and ophthalmoplegia, and hypoesthesia involving the ophthalmic and maxillary divisions of the right trigeminal nerve. MRI demonstrated focal thickening and gadolinium enhancement of the right cavernous sinus together with right extraocular muscle involvement. Following an extensive investigation that did not identify a more compelling infectious, vascular, autoimmune, neoplastic, or neuromuscular etiology, THS was considered the most likely diagnosis. High-dose IV methylprednisolone resulted in rapid resolution of retro-orbital pain and progressive recovery of ptosis and ocular motility, with subsequent radiological improvement. However, prolonged corticosteroid therapy, continued for approximately one year, was associated with significant deterioration of glycemic control requiring insulin treatment and intensification of antihypertensive therapy. This case illustrates both the diagnostic complexity of THS and the importance of longitudinal care beyond neurological recovery. Close collaboration between primary and secondary care was essential for monitoring clinical evolution, managing corticosteroid-related complications, optimizing chronic comorbidities, and coordinating long-term multidisciplinary follow-up.

## Introduction

Tolosa-Hunt syndrome (THS) is a rare neuro-ophthalmological disorder characterized by unilateral orbital or periorbital pain associated with paresis of one or more ocular motor cranial nerves, resulting from idiopathic granulomatous inflammation involving the cavernous sinus, superior orbital fissure, or orbit [1-3]. Its true incidence and prevalence remain uncertain because of its rarity and the limited availability of population-based data. Clinical manifestations commonly include retro-orbital or periorbital headache, diplopia, ptosis, and restricted ocular movements, with involvement of the oculomotor, trochlear, and abducens nerves. The ophthalmic and maxillary divisions of the trigeminal nerve may also be affected because of their anatomical relationship with the cavernous sinus [2-4].

Diagnosis remains challenging because no single clinical, laboratory, or radiological finding is specific for THS. According to the International Classification of Headache Disorders, Third Edition (ICHD-3), diagnosis requires unilateral orbital or periorbital headache associated with paresis of one or more of the ipsilateral third, fourth, and/or sixth cranial nerves and demonstration of granulomatous inflammation by MRI or biopsy. The headache must precede the ocular motor paresis by ≤2 weeks or develop concurrently with it [1]. Contrast-enhanced MRI is the principal imaging modality for evaluating the cavernous sinus and adjacent structures, while laboratory investigations and additional imaging are directed toward excluding alternative causes [1,2,5]. The differential diagnosis of painful ophthalmoplegia is broad and includes vascular disorders, infectious processes, neoplasms, idiopathic orbital inflammatory disease, systemic inflammatory or granulomatous disorders, and other neurological causes [2,5].

Corticosteroids remain the mainstay of treatment and typically result in rapid improvement of orbital pain, although recovery of cranial nerve dysfunction may occur more gradually [2-4]. Nevertheless, corticosteroid responsiveness is supportive rather than specific for THS, and recurrence remains an important consideration during long-term follow-up [2,4,5]. Prolonged corticosteroid exposure may also result in clinically significant adverse effects, particularly in patients with preexisting metabolic or cardiovascular comorbidities. This report describes a diagnostically challenging case of THS with cavernous sinus and extraocular muscle involvement and highlights both the difficulties of establishing the diagnosis and the complexities of prolonged corticosteroid management, including the role of primary care in longitudinal follow-up and management of treatment-related complications.

## Case presentation

A 59-year-old woman with a medical history of type 2 diabetes mellitus, arterial hypertension, dyslipidemia, and post-thyroidectomy hypothyroidism presented to her family physician with a one-day history of retro-orbital headache and blurred vision. Her diabetes had previously been controlled with metformin alone, with a baseline glycated hemoglobin (HbA1c) of 6.2%. A comprehensive neurological examination was unremarkable, with no focal neurological deficits identified. Given the nature of her symptoms, a non-contrast head CT scan was requested.

Three days after symptom onset, the headache and blurred vision persisted, and the patient developed diplopia, prompting presentation to the ED. She was assessed by the internal medicine team, and a non-contrast head CT scan showed no acute intracranial abnormalities. Following discussion with the neurology team, conservative management was recommended. Given the patient’s vascular risk factors and the diagnostic possibility of a vascular cause for the new ocular symptoms, acetylsalicylic acid 100 mg daily was initiated while further neurological evaluation was pursued.

Twenty-five days after the initial presentation, the patient returned to the ED because of progressive neurological deterioration. Physical examination revealed complete right-sided ptosis, complete right ophthalmoplegia, hypoesthesia of the right hemiface, and flattening of the right nasolabial fold. Because timely access to MRI within the public healthcare setting was limited, the patient underwent contrast-enhanced brain MRI at a private imaging center at her own expense. MRI demonstrated moderate thickening and enhancement of the right extraocular muscles, predominantly involving the medial rectus, together with focal thickening and gadolinium enhancement of the anterior portion of the right cavernous sinus (Figure 1A, 1B). These findings raised the differential diagnosis of an inflammatory process involving the orbit and cavernous sinus, including idiopathic orbital inflammatory disease and THS.

**Figure 1:**
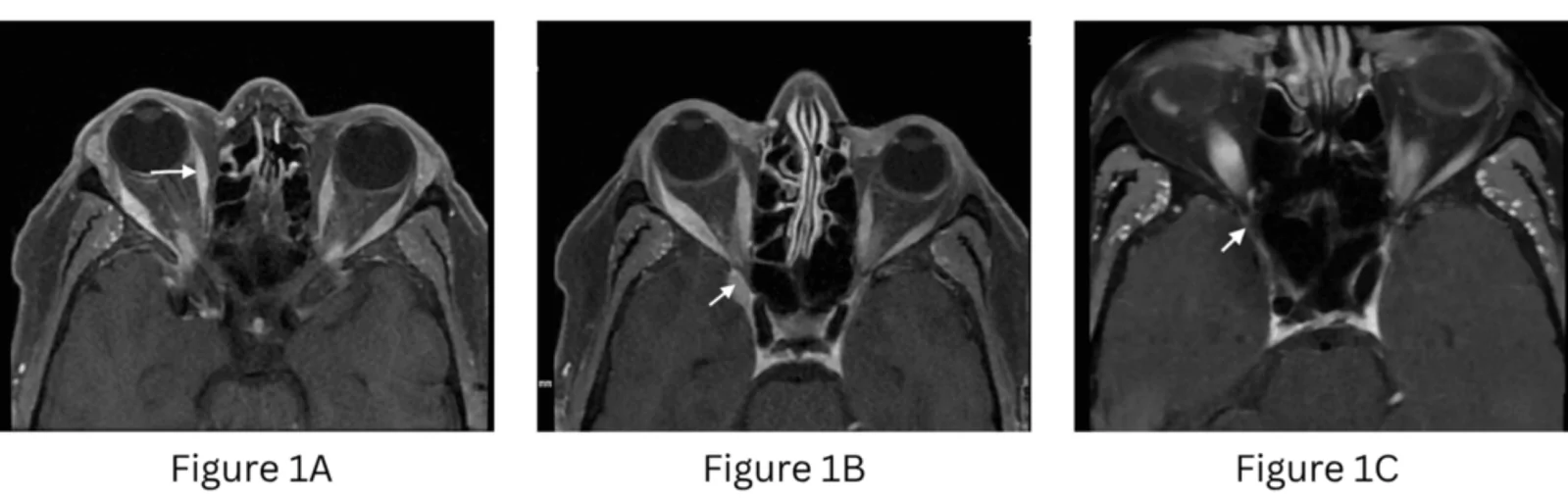
Contrast-enhanced axial MRI demonstrating (A) thickening and enhancement of the right extraocular muscles, predominantly involving the medial rectus (arrow); (B) focal thickening and enhancement of the anterior portion of the right cavernous sinus at initial presentation (arrow); and (C) follow-up imaging demonstrating reduction in the thickening and enhancement of the right cavernous sinus, with residual inflammatory activity (arrow).

Following review of the MRI findings and further discussion with the neurology team, the patient was admitted to the neurology ward for further investigation. Neurological examination confirmed complete right-sided ptosis and complete right ophthalmoplegia, with additional limitation of upward gaze in the left eye. Sensory examination demonstrated hypoesthesia in the territories of the ophthalmic (V1) and maxillary (V2) divisions of the right trigeminal nerve. Flattening of the right nasolabial fold was again documented. An ophthalmology assessment was requested; however, the corresponding consultation report was not available for review. The limitation of upward gaze in the left eye was not documented again in the available records or during subsequent follow-up assessments. Therefore, persistence or resolution of this finding could not be established.

A broad diagnostic workup was undertaken to investigate alternative infectious, autoimmune, vascular, neoplastic, and neuromuscular causes of painful ophthalmoplegia. CSF analysis showed three cells/µL (100% mononuclear), mildly elevated protein at 45 mg/dL, and glucose of 93 mg/dL, without significant pleocytosis. Serum glucose measured on the same day was 140 mg/dL, corresponding to a CSF-to-serum glucose ratio of approximately 0.66. The erythrocyte sedimentation rate was elevated at 48 mm/h and subsequently increased to 81 mm/h. Serological testing for human immunodeficiency virus, hepatitis B and C viruses, and syphilis was negative. Magnetic resonance angiography demonstrated preserved morphology and caliber of the major intracranial arteries, without relevant vascular abnormalities. Thoracic CT showed no pulmonary nodules, parenchymal consolidation, fibrotic changes, or mediastinal or hilar lymphadenopathy suggestive of an underlying thoracic granulomatous or neoplastic process. Additional autoimmune, infectious, and neuromuscular investigations were requested, some of which remained pending during hospitalization. The principal investigations performed during the diagnostic workup are summarized in Table 1.

**Table 1:** Diagnostic workup performed during the evaluation of painful ophthalmoplegia. AMA-M2, anti-mitochondrial M2 antibodies; ANA, antinuclear antibodies; ANCA, antineutrophil cytoplasmic antibodies; anti-dsDNA, anti-double-stranded DNA antibodies; DFS70, dense fine speckled 70; EMG, electromyography; HBV, hepatitis B virus; HCV, hepatitis C virus; IGRA, interferon-gamma release assay; MPO, myeloperoxidase; p-ANCA, perinuclear antineutrophil cytoplasmic antibodies; PR3, proteinase 3; Ro52, tripartite motif-containing protein 21 (TRIM21); SSA, Sjögren’s syndrome-related antigen A

Investigation	Result	Diagnostic relevance
Non-contrast head CT	No acute intracranial abnormalities	No acute structural intracranial cause identified
Contrast-enhanced brain MRI	Thickening and enhancement of the right extraocular muscles, predominantly the medial rectus; focal thickening and enhancement of the anterior right cavernous sinus	Supported an inflammatory process involving the orbit and cavernous sinus
Magnetic resonance angiography	No relevant abnormalities of the major intracranial arteries	No major intracranial arterial abnormality identified
CSF	3 cells/µL (100% mononuclear); protein 45 mg/dL; glucose 93 mg/dL; same-day serum glucose 140 mg/dL (CSF/serum glucose ratio ≈0.66)	No significant cerebrospinal fluid inflammatory abnormalities
Repetitive nerve stimulation/electromyography	No abnormalities	Did not support a neuromuscular junction disorder
HIV, HBV, HCV, and *Treponema pallidum* testing	Negative	No evidence supporting these infectious etiologies
*Borrelia burgdorferi* testing	Negative	No evidence supporting Lyme disease
IGRA	Negative	No evidence supporting tuberculosis infection
Thoracic CT	No pulmonary lesions or relevant thoracic lymphadenopathy	No radiological findings supporting thoracic granulomatous or neoplastic disease
Anti-dsDNA	Negative	Did not support systemic lupus erythematosus
ANA	Negative nuclear staining; granular cytoplasmic pattern present	Required correlation with clinical and additional immunological findings
Immunoblot autoimmune testing	Reactivity for DFS70, AMA-M2, Ro52 (TRIM21), and SSA antibodies	Findings required clinical correlation and did not independently establish an alternative systemic diagnosis
Erythrocyte sedimentation rate	48 mm/h, subsequently 81 mm/h	Elevated inflammatory marker
Serum ACE	11 U/L (within reference range)	No serum ACE elevation supporting sarcoidosis
Serum calcium	9.1 mg/dL (within reference range; subsequent testing)	No hypercalcemia identified
ANCA testing	Atypical p-ANCA positive; MPO and PR3 antibodies negative	Did not support a typical ANCA-associated vasculitis

Before the MRI findings were available, a neuromuscular junction disorder was considered as a possible explanation for the prominent ocular motor abnormalities. Repetitive nerve stimulation/electromyography was therefore requested, and empirical IVIG at 30 g/day, initially planned for four days, was initiated while the diagnostic investigation was ongoing. However, the subsequent MRI findings demonstrating inflammatory abnormalities involving the right cavernous sinus shifted the diagnostic reasoning away from a neuromuscular junction disorder and toward an inflammatory cavernous sinus process, and IVIG was discontinued. Repetitive nerve stimulation subsequently showed no abnormalities, providing no electrophysiological support for a neuromuscular junction disorder. Integration of the progressive painful ophthalmoplegia, involvement of multiple cranial nerves, characteristic cavernous sinus abnormalities on MRI, and absence of a more compelling alternative etiology ultimately supported a working diagnosis of THS.

Further immunological investigation showed normal serum angiotensin-converting enzyme (11 U/L), negative anti-double-stranded DNA antibodies, normal C3, mildly reduced C4, and negative rheumatoid factor. Antinuclear antibody testing by immunofluorescence showed negative nuclear staining with a granular cytoplasmic pattern, while immunoblot testing demonstrated reactivity for DFS70, AMA-M2, Ro52, and SSA antibodies. Subsequent antineutrophil cytoplasmic antibody (ANCA) testing demonstrated an atypical p-ANCA pattern, with negative myeloperoxidase (MPO) and PR3 antibodies. Given the Ro52/SSA and AMA-M2 reactivities, rheumatology follow-up was pursued to investigate a possible underlying systemic autoimmune disorder. However, no alternative systemic autoimmune diagnosis was established during follow-up. Liver biochemistry remained within the reference range, including aspartate aminotransferase, alanine aminotransferase, alkaline phosphatase, and gamma-glutamyl transferase.

High-dose IV methylprednisolone was subsequently initiated at 1 g once daily for five consecutive days. The patient demonstrated a rapid clinical response, with resolution of the retro-orbital pain, marked improvement in right-sided ptosis, and progressive recovery of right ocular motility in both the horizontal and vertical planes.

Following initiation of high-dose corticosteroid therapy, the patient developed significant hyperglycemia due to decompensation of her preexisting type 2 diabetes mellitus, prompting reassessment by the internal medicine team. Intermediate-acting insulin was initiated, with subsequent improvement in glycemic control. After completing five days of IV methylprednisolone, the patient was discharged on oral prednisolone 60 mg once daily and intermediate-acting insulin 6 IU twice daily. Follow-up was arranged with neurology, the diabetes clinic, and her family physician for corticosteroid tapering, monitoring of neurological recovery and glycemic control, and management of treatment-related complications.

During subsequent outpatient follow-up, marked neurological improvement was documented. Right-sided ptosis resolved, facial sensation normalized, and right ocular motility recovered substantially, although residual diplopia persisted. Results from investigations requested during hospitalization also became available and were assessed in the context of the overall clinical presentation. The immunological findings did not independently establish an alternative systemic autoimmune diagnosis, and further assessment by rheumatology was arranged.

The prolonged corticosteroid course required close metabolic monitoring. Glycemic control deteriorated despite insulin therapy, with HbA1c increasing from 6.2% before corticosteroid treatment to 8.1%. Insulin doses were progressively adjusted according to home capillary glucose measurements. Arterial hypertension also became more difficult to control and required intensification of antihypertensive therapy with perindopril 10 mg, amlodipine 5 mg, and nebivolol 5 mg. The marked TSH elevation was interpreted in the context of her preexisting post-thyroidectomy hypothyroidism rather than attributed directly to corticosteroid therapy, and levothyroxine treatment was subsequently adjusted. These comorbidities were monitored longitudinally in primary care in coordination with the diabetes and neurology teams.

Corticosteroid therapy was gradually tapered according to clinical and radiological evolution, from the initial prednisolone dose of 60 mg/day to 20 mg/day and subsequently to 15 and 10 mg/day. Follow-up MRI demonstrated a reduction in the volume and enhancement of the affected portion of the right cavernous sinus, although residual inflammatory activity persisted (Figure 1C). Overall, corticosteroid therapy was continued for approximately one year and was ultimately discontinued after tapering to a final dose of 5 mg/day, with the prolonged taper guided by the clinical course and residual radiological activity.

At subsequent follow-up, the patient remained free of retro-orbital pain and ptosis, with normal facial sensation and substantial recovery of ocular motility. Residual diplopia persisted, particularly on right gaze, although ocular occlusion was no longer routinely required. She was subsequently assessed by ophthalmology and rheumatology, while neurology continued to supervise the corticosteroid taper. Glycemic control progressively improved with continued adjustment of antidiabetic therapy, with subsequent HbA1c values ranging from 6.1% to 6.5%. Continued follow-up by her family physician provided longitudinal monitoring of neurological symptoms, glycemic control, blood pressure, thyroid function, and treatment-related adverse effects throughout the prolonged corticosteroid course.

Given the anticipated prolonged corticosteroid exposure, calcium and vitamin D supplementation and omeprazole were initiated at hospital discharge. Bone mineral density assessment by dual-energy X-ray absorptiometry (DXA) was not performed.

The chronology of the clinical presentation, diagnostic evaluation, treatment, and subsequent follow-up is summarized in Figure 2.

**Figure 2:**
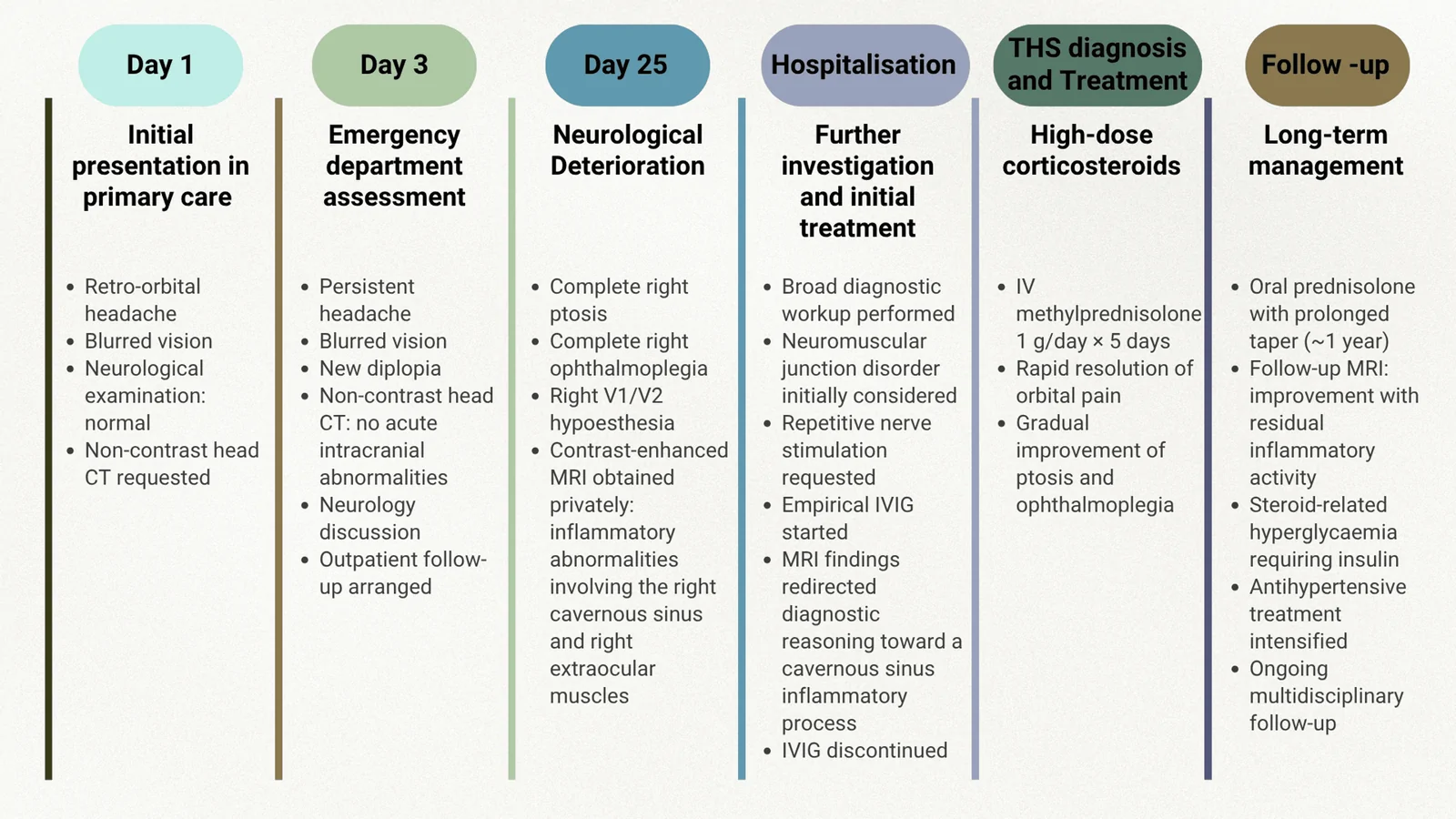
Timeline of clinical presentation, diagnostic evaluation, treatment, and follow-up. THS, Tolosa-Hunt syndrome This figure was created using Canva (Canva Pty Ltd., Sydney, Australia).

## Discussion

This case describes a 59-year-old woman who presented with progressive painful ophthalmoplegia and was ultimately diagnosed with THS following an extensive investigation for alternative infectious, vascular, autoimmune, neoplastic, and inflammatory causes. The diagnosis was supported by the clinical course, MRI abnormalities involving the right cavernous sinus, and rapid response to high-dose corticosteroid therapy. In addition to the diagnostic challenges associated with this rare condition, this case illustrates the importance of longitudinal follow-up, particularly in patients with preexisting comorbidities who develop complications related to prolonged corticosteroid treatment.

Our patient’s presentation closely mirrors the classical clinical course of THS, in which unilateral orbital or retro-orbital pain typically precedes or occurs concurrently with ophthalmoplegia. Cranial nerves III, IV, and VI are most frequently affected, while involvement of the ophthalmic (V1) and maxillary (V2) divisions of the trigeminal nerve may also occur because of their anatomical relationship with the cavernous sinus [2-5]. In our patient, retro-orbital headache preceded the development of ophthalmoplegia, ptosis, and sensory impairment involving the V1 and V2 territories, providing a clinically coherent anatomical pattern of cavernous sinus involvement. The combination of unilateral painful ophthalmoplegia, involvement of multiple ocular motor cranial nerves, trigeminal sensory impairment involving V1 and V2, and corresponding inflammatory abnormalities of the cavernous sinus on contrast-enhanced MRI constituted the principal clinical and radiological features supporting THS in this patient [2,3,5].

When considered against the ICHD-3 diagnostic criteria, our patient demonstrated several characteristic features of THS, including unilateral retro-orbital pain preceding ophthalmoplegia, ipsilateral ocular motor dysfunction, and MRI evidence of an inflammatory process involving the right cavernous sinus [1]. The ICHD-3 criteria require demonstration of granulomatous inflammation by MRI or biopsy, together with paresis of one or more of the ipsilateral third, fourth, and/or sixth cranial nerves, with the headache preceding the ocular motor paresis by ≤2 weeks or developing with it [1]. In our patient, retro-orbital headache began on day 1 and diplopia developed by day 3, providing clinical evidence of ocular motor involvement within the two-week interval specified by ICHD-3, although complete right ophthalmoplegia was only documented by day 25. Histopathological confirmation was not obtained in our patient; however, biopsy is not mandatory when compatible MRI findings are present and alternative etiologies have been appropriately investigated [1,2]. Given the anatomical location of the lesion, the invasive nature of biopsy, the concordant clinical and radiological findings, and the favorable therapeutic response, histopathological confirmation was not pursued.

The initial presentation also illustrates an important diagnostic challenge in primary care. At her first assessment, the patient reported retro-orbital headache and blurred vision but had no identifiable focal neurological deficit. Pain may precede ophthalmoplegia by several days in THS, meaning that the earliest presentation can be nonspecific and may not initially fulfill the expected clinical picture [1-3,5]. However, the development of diplopia by day 3 represented an important change in the clinical presentation. Although non-contrast CT showed no acute intracranial abnormality, it did not identify the inflammatory cavernous sinus process subsequently demonstrated on contrast-enhanced MRI. In retrospect, earlier contrast-enhanced MRI following the development of painful diplopia would have been diagnostically valuable and might have shortened the 25-day interval to diagnosis. In this case, however, timely access to MRI within the public healthcare setting was limited, and the patient ultimately obtained the examination at a private imaging center at her own expense. The subsequent progression to multiple cranial neuropathies highlights the importance of clinical reassessment and escalation of neuroimaging when painful ophthalmoplegia persists or progresses despite initially unrevealing CT findings.

An unusual finding in this patient was flattening of the right nasolabial fold, raising the possibility of facial nerve involvement. The facial nerve does not traverse the cavernous sinus, and its involvement is therefore not characteristic of the typical anatomical pattern of THS. Nevertheless, facial nerve involvement has occasionally been reported in association with THS [5]. The significance of this finding in our patient remains uncertain and may have represented subtle facial weakness, preexisting facial asymmetry, or an unrelated examination finding. This reinforces the importance of cautious interpretation of neurological findings that cannot readily be explained by the anatomical site of the suspected lesion.

Another atypical finding was limitation of upward gaze in the left eye during the inpatient neurological examination. THS is characteristically unilateral, and a contralateral ocular motor abnormality would broaden the differential diagnosis and raise consideration of alternative or more diffuse processes [2,5]. In this case, the left-sided finding was documented on a single examination and was not mentioned again in the available subsequent clinical records. Although an ophthalmology assessment was requested, the corresponding consultation report was not available for review; therefore, it was not possible to determine whether this finding was confirmed or subsequently resolved. In contrast, the predominant neurological abnormalities and MRI findings remained right-sided. This isolated finding therefore remains an area of diagnostic uncertainty and reinforces the importance of longitudinal reassessment and consideration of alternative diagnoses.

MRI is the imaging modality of choice when THS is suspected. Typical abnormalities include enlargement and contrast enhancement of the cavernous sinus, with possible extension through the superior orbital fissure into the orbital apex [2,3,5-7]. Similar findings have been documented in dedicated radiological reports of THS, and serial MRI may demonstrate partial or complete regression of these abnormalities following corticosteroid therapy [6,7]. In our patient, MRI demonstrated focal thickening and gadolinium enhancement of the anterior portion of the right cavernous sinus, together with thickening and enhancement of the right extraocular muscles. Although these findings supported an inflammatory process involving the cavernous sinus, MRI abnormalities in THS are not pathognomonic. Similar imaging appearances may occur in other inflammatory, infectious, and neoplastic conditions; therefore, MRI findings must be interpreted in conjunction with the clinical presentation and appropriate exclusion of alternative diagnoses [2,3,5,6].

The presence of extraocular muscle thickening was particularly relevant because it broadened the radiological differential diagnosis to include idiopathic orbital inflammatory disease. Radiological overlap between THS and orbital inflammatory disease has been recognized, particularly when inflammatory changes extend into the orbit and involve the extraocular muscles [5,8]. In our patient, however, the concomitant cavernous sinus involvement, progressive involvement of multiple cranial nerves, and overall clinical course favored THS. This illustrates the importance of correlating radiological findings with the clinical phenotype rather than establishing the diagnosis from imaging alone [5].

THS remains fundamentally a diagnosis of exclusion. No specific biochemical or radiological marker independently establishes the diagnosis, and limitations of the current diagnostic criteria contribute to persistent diagnostic uncertainty [5,9,10]. The differential diagnosis of painful ophthalmoplegia is broad and includes vascular disorders such as intracranial aneurysm and cavernous sinus thrombosis; infectious processes involving the orbit or cavernous sinus; neoplastic lesions; systemic inflammatory and granulomatous diseases, including sarcoidosis, granulomatosis with polyangiitis, systemic lupus erythematosus, and IgG4-related disease; and other neurological conditions such as diabetic cranial neuropathy [2,3,5,9,10]. THS-like presentations have also been reported in association with systemic disorders such as sarcoidosis, further illustrating the importance of investigating secondary causes before accepting an idiopathic diagnosis [11]. This distinction is clinically important because several THS mimics may produce similar clinical and radiological findings, and corticosteroid treatment may obscure an alternative diagnosis or exacerbate an unrecognized infectious process [5,9,10]. Mullen et al. demonstrated clinically relevant false-positive and false-negative classifications when applying the ICHD-3 criteria, further emphasizing the need for careful exclusion of alternative etiologies and continued follow-up [9].

A neuromuscular junction disorder was also initially considered because of the prominent ocular motor abnormalities, prompting repetitive nerve stimulation and empirical IVIG before the MRI results became available. However, this hypothesis became less compelling when the overall clinical pattern, including retro-orbital pain and trigeminal sensory impairment, was considered alongside the subsequent demonstration of inflammatory abnormalities involving the cavernous sinus on MRI. The evolving clinical and radiological findings therefore redirected the diagnostic approach toward an inflammatory cavernous sinus process.

In our patient, the diagnostic workup did not identify an alternative condition that adequately explained the overall presentation. Vascular causes were investigated by magnetic resonance angiography, which did not demonstrate a relevant vascular lesion. Cerebrospinal fluid analysis showed 3 cells/µL, mildly elevated protein (45 mg/dL), and glucose of 93 mg/dL; same-day serum glucose was 140 mg/dL, corresponding to a CSF-to-serum glucose ratio of approximately 0.66. Investigations for HIV, hepatitis B and C, syphilis, *Borrelia* infection, and tuberculosis were unrevealing. The erythrocyte sedimentation rate was elevated at 48 mm/h and subsequently increased to 81 mm/h. Thoracic imaging showed no pulmonary lesions or relevant lymphadenopathy, and serum angiotensin-converting enzyme was within the reference range (11 U/L); serum calcium was also within the reference range on subsequent testing. ANCA testing performed during follow-up demonstrated an atypical p-ANCA pattern, with negative MPO and PR3 antibodies. Although serum IgG4 was not measured and CSF cytology was not performed, the overall investigation did not identify a more compelling infectious, vascular, systemic inflammatory, or neoplastic explanation for the clinical and radiological presentation.

The immunological findings required cautious interpretation. Ro52, SSA, and AMA-M2 reactivities prompted consideration of systemic autoimmune disease, including Sjögren syndrome and primary biliary cholangitis. However, subsequent rheumatology evaluation did not establish a systemic autoimmune disease that better explained the neurological presentation; liver biochemistry remained unremarkable, and a subsequent minor salivary gland biopsy showed no inflammatory infiltrate or other pathological abnormalities. Anti-DFS70 antibodies have been associated with a lower likelihood of systemic autoimmune rheumatic disease, particularly when present in isolation without other disease-associated autoantibodies [12]. In this patient, however, the concomitant Ro52, SSA, and AMA-M2 reactivities limited the exclusionary significance of the anti-DFS70 finding [12]. Continued clinical follow-up was therefore considered appropriate.

Diabetic cranial neuropathy represented a particularly relevant alternative diagnosis given the patient’s history of type 2 diabetes mellitus. However, an isolated diabetic ocular motor neuropathy would not adequately account for the overall presentation, particularly the involvement of multiple cranial nerves, sensory abnormalities in the V1 and V2 territories, and inflammatory abnormalities involving the cavernous sinus on MRI. Taken together, the clinical and radiological findings favored a cavernous sinus inflammatory process rather than an isolated diabetic cranial neuropathy [3,5].

Corticosteroids remain the mainstay of treatment for THS. Orbital pain typically improves rapidly following treatment initiation, often within 24-72 hours, whereas recovery of cranial nerve dysfunction may occur more gradually over subsequent weeks [3,4]. In our patient, IV methylprednisolone resulted in rapid resolution of retro-orbital pain, followed by progressive improvement in ptosis and ophthalmoplegia. Follow-up MRI additionally demonstrated a reduction in the volume and enhancement of the affected portion of the right cavernous sinus, providing radiological evidence of improvement alongside the clinical response. Similar partial or complete regression of cavernous sinus abnormalities following systemic corticosteroid therapy has been demonstrated on serial MRI in THS [13].

For patients with recurrent, steroid-dependent, or refractory THS, steroid-sparing immunosuppressive therapy may be considered, although the available evidence is limited and is derived largely from retrospective studies and case reports [14,15]. Agents including azathioprine, methotrexate, and mycophenolate mofetil have been used as steroid-sparing or second-line therapies [14,15]. Such strategies may be particularly relevant when prolonged corticosteroid exposure results in substantial treatment-related toxicity. In our patient, steroid-sparing therapy was not required; however, the prolonged treatment course and deterioration of glycemic control illustrate why such an approach might be considered if recurrent disease, steroid dependence, or inability to achieve corticosteroid withdrawal were to occur.

Importantly, response to corticosteroids should not be regarded as diagnostic of THS in isolation. Other inflammatory and neoplastic conditions may also demonstrate clinical improvement following corticosteroid treatment [5]. Furthermore, limitations of the current diagnostic criteria have been demonstrated. Mullen et al. reported both false-positive and false-negative classifications when applying the ICHD-3 criteria, emphasizing the need for continued clinical and radiological surveillance even when the initial presentation appears compatible with THS [9]. More recently, Chądzyński et al. evaluated six patients with painful ophthalmoplegia and found that only one fulfilled the complete ICHD-3 criteria for THS, despite all six responding successfully to corticosteroid therapy [10]. This further illustrates why therapeutic response should be considered supportive rather than diagnostic. Premature attribution of painful ophthalmoplegia to THS may delay recognition of an alternative underlying disease, and, in the case of an infectious etiology, corticosteroid therapy may exacerbate the underlying condition [9,10]. Serial imaging may therefore provide additional diagnostic information, particularly when the clinical or radiological response is incomplete or atypical; notably, Çakırer reported a presumed THS case in which an inadequate response prompted reconsideration of the diagnosis, and the cavernous sinus lesion was subsequently identified as a meningioma [13].

Long-term follow-up is additionally important because THS may relapse. A 2026 systematic review and meta-analysis of 17 studies comprising 456 patients reported a pooled recurrence rate of 23% (95% CI, 17-32%), indicating that approximately one in four patients experiences recurrence [16]. This substantial recurrence rate supports continued clinical surveillance during and after corticosteroid withdrawal.

This case also illustrates that successful management of THS may extend well beyond neurological recovery. In our patient, corticosteroid therapy continued for approximately one year, with a prolonged taper to 5 mg/day before eventual discontinuation. Given the anticipated prolonged corticosteroid exposure, calcium and vitamin D supplementation and gastroprotection with omeprazole were instituted; however, bone mineral density assessment by DXA was not performed. Before corticosteroid treatment, her type 2 diabetes mellitus was controlled with metformin alone, with an HbA1c of 6.2%. During corticosteroid therapy, glycemic control deteriorated substantially, requiring insulin treatment, with HbA1c subsequently increasing to 8.1%. Her arterial hypertension also required intensification of pharmacological therapy. As the corticosteroid dose was progressively reduced and antidiabetic treatment adjusted, glycemic control improved. The family physician therefore played an important longitudinal role in monitoring corticosteroid-related complications, adjusting treatment for chronic comorbidities, assessing persistent neurological symptoms, and coordinating multidisciplinary follow-up with neurology, diabetes, ophthalmology, and rheumatology.

Several limitations and areas of diagnostic uncertainty should be acknowledged. Histopathological confirmation was not obtained, and the extraocular muscle abnormalities observed on MRI overlap with those described in idiopathic orbital inflammatory disease. Although an extensive investigation for alternative etiologies was performed, the diagnostic workup was not exhaustive; serum IgG4 testing and CSF cytology were not performed. In addition, the significance of the finding suggestive of facial nerve involvement, the isolated limitation of upward gaze in the contralateral eye, and the immunological abnormalities detected during follow-up remain uncertain. Finally, a favorable corticosteroid response is supportive but not specific for THS. Nevertheless, the predominant anatomical pattern of cranial nerve involvement, inflammatory abnormalities involving the cavernous sinus on MRI, exclusion of several important alternative etiologies, and concordant clinical and radiological improvement collectively favored THS as the most likely diagnosis [2,5,9,10].

Taken together, this case demonstrates that the diagnosis of THS should not rely on any single clinical feature, MRI abnormality, or response to corticosteroid treatment. Rather, diagnosis requires integration of the anatomical pattern of cranial nerve involvement, appropriate neuroimaging, systematic exclusion of potentially serious mimics, therapeutic response, and longitudinal clinical and radiological follow-up [1,2,5,9,10]. The case additionally highlights the role of primary care both at the initial, potentially nonspecific stage of presentation and during prolonged follow-up, when management of corticosteroid-related complications and coordination of multidisciplinary care become central to patient care.

## Conclusions

THS is a rare but important cause of painful ophthalmoplegia that requires careful exclusion of vascular, infectious, inflammatory, and neoplastic conditions. Diagnosis relies on integration of the clinical presentation, compatible imaging findings, and systematic exclusion of alternative etiologies, rather than on any single clinical, radiological, or therapeutic feature. Once the diagnosis is established, corticosteroid therapy is generally associated with rapid improvement in pain, although neurological recovery may be more gradual. Long-term follow-up remains important because of the potential for recurrence.

This case further demonstrates that the management of THS extends beyond neurological recovery, particularly in patients with comorbidities who may be vulnerable to complications of prolonged corticosteroid therapy. Close collaboration between primary and secondary care is therefore important, with the family physician playing a central role in longitudinal monitoring, management of treatment-related complications, optimization of chronic comorbidities, and coordination of multidisciplinary care.
